# Supplementary material for: Laboratory-confirmed respiratory viral infection triggers for acute myocardial infarction and stroke: Systematic review protocol
Source: PLoS One. 2024 Jul 10;19(7):e0302748. doi: 10.1371/journal.pone.0302748 (PMC11236192; doi:10.1371/journal.pone.0302748)
Supplement: S2 File — Search strategy for Ovid MEDLINE, Ovid Embase, PubMed, Cochrane Central Register of Controlled Trials (CENTRAL), and Web of Science. (DOCX) [file pone.0302748.s002.docx]

**S2 File. Search strategy.** Full search strategy for MEDLINE, EMBASE, PubMed, Cochrane Central Register of Controlled Trials, and Web of Science

**MEDLINE (via Ovid)**
1. (Lab* adj2 (test* or confirm* or result* or detect* or diagnos*)).tw,kf.

2. exp Polymerase Chain Reaction/

3. (polymerase-chain-reaction* or PCR or multi-plex or multiplex or monoplex).tw,kf.

4. ((Molecular or nucleic-acid or microbiologic*) adj1 (diagnos* or test* or assay*)).tw,kf.

5. exp Serologic Tests/ or exp microbiological techniques/ or exp fluorescent antibody technique/

6. (serum* or serolog* or blood-sample or (sero* adj1 (positive* or negative* or epidemiolog*))).tw,kf.

7. (respiratory-panel* or pathogen-panel* or RP-panel* or respiratory-pathogen*).tw,kf.

8. (((throat* or nasal* or nasopharyngeal or naso-pharyngeal or respiratory) adj2 (swab* or specimen* or sample* or wash*)) or nasopharyngeal-aspirate or naso-pharyngeal-aspirate).tw,kf.

9. (culture* or cultivat* or isolat*).tw,kf.

10. (antigen-detect* or antibody-test* or immunoassay* or immuno-assay* or immunofluorescen* or immuno-fluorescen* or electron-microscopy or ELISA).tw,kf.

11. or/1-10

12. exp Respiratory Tract Infections/

13. (((chest or respiratory or lung) adj2 infect*) or RTI or URTI or LRTI or acute-respiratory or common-cold*).tw,kf.

14. exp *Virus Diseases/ or exp *Viruses/

15. (viral* or virus* or virology).tw,kf.

16. exp influenzavirus a/ or exp influenzavirus b/ or Influenza, Human/

17. (influenza* or flu).tw,kf.

18. exp Coronaviridae/ or exp coronavirus infections/

19. (SARS-CoV-2 or covid* or coronavirus* or severe-acute-respiratory-syndrome* or SARS or Middle-East-respiratory-syndrome* or MERS).tw,kf.

20. exp Picornaviridae/ or exp Picornaviridae Infections/

21. (picornavirus* or rhinovirus*or parechovirus* or echovirus* or enterovirus*).tw,kf.

22. exp Paramyxoviridae/ or exp Paramyxoviridae Infections/

23. Metapneumovirus/ or exp Pneumovirus Infections/

24. (Metapneumovirus* or HMPV).tw,kf.

25. Parainfluenza/ or exp parainfluenza virus 1, human/ or exp parainfluenza virus 3, human/

26. parainfluenza*.tw,kf.

27. Respiratory Syncytial Virus, Human/ or Respiratory Syncytial Virus Infections/

28. (RSV or respiratory-syncytial).tw,kf.

29. exp Adenoviridae/ or exp Adenovirus Infections, Human/

30. (Adenovirus* or adenoviridae).tw,kf.

31. exp Herpesviridae/ or exp Herpesviridae Infections/

32. (herpes* or epstein-barr* or EBV or cytomegalovirus* or CMV or varicella or VZV).tw,kf.

33. Human bocavirus/ or exp Parvoviridae Infections/

34. (human-bocavirus* or HBoV).tw,kf.

35. or/12-34

36. exp Myocardial Infarction/

37. (AMI or MI or STEMI or nSTEMI).tw,kf.

38. ((coronary or cardiovascular or heart or myocardial or cardiac) adj2 (infarct* or isch?emi* or attack*)).tw,kf.

39. ((isch?emi* or thrombo*) adj2 event*).tw,kf.

40. exp Stroke/ or exp Brain Infarction/ or Cerebral Hemorrhage/ or exp Subarachnoid Haemorrhage/

41. (stroke* or ((brain or cerebr* or isch?emi* or h?emorrhag*) adj2 (stroke* or infarct* or failure*))).tw,kf.

42. ((cardiovascular or cerebr*) adj1 (acute or mortalit* or death*)).tw,kf.

43. or/36-42

44. randomized controlled trial.pt.

45. (random$ or placebo$ or single blind$ or double blind$ or triple blind$).ti,ab.

46. (retraction of publication or retracted publication).pt.

47. or/44-46

48. ((comment or editorial or meta-analysis or practice-guideline or review or letter) not "randomized controlled trial").pt.

49. (random sampl$ or random digit$ or random effect$ or random survey or random regression).ti,ab. not "randomized controlled trial".pt.

50. 47 not (48 or 49)

51. exp cohort studies/

52. cohort$.tw.

53. controlled clinical trial.pt.

54. epidemiologic methods/

55. limit 54 to yr=1966-1989

56. exp case-control studies/

57. (case$ and control$).tw.

58. (case$ and (series or crossover or cross-over)).tw.

59. or/51-53,55-58

60. (exp animals/ or (rat or rats or mouse or mice or rodent* or swine or porcine or murine or sheep or lamb or lambs or pig or pigs or piglet or piglets or rabbit or rabbits or cat or cats or dog or dogs or cattle or bovine or monkey or monkeys or trout or marmoset or marmosets).ti.) not human*.sh.

61. 11 and 35 and 43 and (50 or 59)

62. 61 not 60

**PubMed (NLM)**

#1 "Lab test"[tiab:~2] OR "Lab tests"[tiab:~2] OR "Lab testing"[tiab:~2] OR "Lab result"[tiab:~2] OR "Lab results"[tiab:~2] OR "Lab detection"[tiab:~2] OR "Lab detections"[tiab:~2] OR "Lab detect"[tiab:~2] OR "Lab diagnosis"[tiab:~2] OR "Lab diagnosed"[tiab:~2] OR "Lab diagnoses"[tiab:~2] OR "Laboratory test"[tiab:~2] OR "Laboratory tests"[tiab:~2] OR "Laboratory testing"[tiab:~2] OR "Laboratory confirmation"[tiab:~2] OR "Laboratory confirmed"[tiab:~2] OR "Laboratory confirmatory"[tiab:~2] OR "Laboratory confirm"[tiab:~2] OR "Laboratory confirmed"[tiab:~2] OR "Laboratory result"[tiab:~2] OR "Laboratory results"[tiab:~2] OR "Laboratory detect"[tiab:~2] OR "Laboratory detected"[tiab:~2] OR "Laboratory detection"[tiab:~2] OR "Laboratory detections"[tiab:~2] OR "Laboratory diagnosis"[tiab:~2] OR "Laboratory diagnosed"[tiab:~2] OR "Laboratory diagnoses"[tiab:~2]

#2 "polymerase chain-reaction*"[tiab] OR “PCR”[tiab] OR "multi plex"[tiab] OR “multiplex”[tiab] OR “monoplex”[tiab]

#3 "Molecular diagnosis"[tiab:~1] OR "Molecular diagnoses"[tiab:~1] OR "Molecular diagnosed"[tiab:~1] OR "Molecular diagnostic"[tiab:~1] OR "Molecular test"[tiab:~1] OR "Molecular tests"[tiab:~1] OR "Molecular testing"[tiab:~1] OR "Molecular tested"[tiab:~1] OR "Molecular assay"[tiab:~1] OR "Molecular assays"[tiab:~1] OR "Nucleic acid diagnosis"[tiab:~1] OR "Nucleic acid diagnoses"[tiab:~1] OR "Nucleic acid diagnosed"[tiab:~1] OR "Nucleic acid diagnostic"[tiab:~1] OR "Nucleic acid test"[tiab:~1] OR "Nucleic acid tests"[tiab:~1] OR "Nucleic acid testing"[tiab:~1] OR "Nucleic acid tested"[tiab:~1] OR "Nucleic acid assay"[tiab:~1] OR "Nucleic acid assays"[tiab:~1] OR "Microbiologic diagnosis"[tiab:~1] OR "Microbiologic diagnoses"[tiab:~1] OR "Microbiologic diagnosed"[tiab:~1] OR "Microbiologic diagnostic"[tiab:~1] OR "Microbiologic test"[tiab:~1] OR "Microbiologic tests"[tiab:~1] OR "Microbiologic testing"[tiab:~1] OR "Microbiologic tested"[tiab:~1] OR "Microbiologic assay"[tiab:~1] OR "Microbiologic assays"[tiab:~1] OR "Microbiological diagnosis"[tiab:~1] OR "Microbiological diagnoses"[tiab:~1] OR "Microbiological diagnosed"[tiab:~1] OR "Microbiological diagnostic"[tiab:~1] OR "Microbiological test"[tiab:~1] OR "Microbiological tests"[tiab:~1] OR "Microbiological testing"[tiab:~1] OR "Microbiological tested"[tiab:~1] OR "Microbiological assay"[tiab:~1] OR "Microbiological assays"[tiab:~1]

#4 “Serum”[tiab] OR “Serological”[tiab] OR “Serology”[tiab] OR “Serologic”[tiab] OR "blood sample"[tiab] OR “Seroepidemiology”[tiab] OR “Seroepidemiological”[tiab] OR "Sero positive"[tiab:~1] OR "Sero negative"[tiab:~1] OR "Sero epidemiology"[tiab] OR "Sero epidemiological"[tiab] OR "Seroepidemiologic"[tiab] OR "Sero epidemiologic"[tiab]

#5 "respiratory panel*"[tiab] OR "pathogen panel*"[tiab] OR "RP panel*"[tiab] OR "respiratory pathogen*"[tiab]

#6 "Throat swab"[tiab:~2] OR "Throat swabbing" [tiab:~2] OR "Throat swabs"[tiab:~2] OR "Throat specimen"[tiab:~2] OR "Throat specimens"[tiab:~2] OR "Throat sample"[tiab:~2] OR "Throat samples"[tiab:~2] OR "Throat wash"[tiab:~2] OR "Throat washing"[tiab:~2] OR "Throat washes"[tiab:~2] OR "Nasal swab"[tiab:~2] OR "Nasal swabbing"[tiab:~2] OR "Nasal swabs"[tiab:~2] OR "Nasal specimen"[tiab:~2] OR "Nasal specimens"[tiab:~2] OR "Nasal sample"[tiab:~2] OR "Nasal samples"[tiab:~2] OR "Nasal wash"[tiab:~2] OR "Nasal washing"[tiab:~2] OR "Nasal washes" OR "Nasopharyngeal swab"[tiab:~2] OR "Nasopharyngeal swabbing"[tiab:~2] OR "Nasopharyngeal swabs"[tiab:~2] OR "Nasopharyngeal specimen"[tiab:~2] OR "Nasopharyngeal specimens"[tiab:~2] OR "Nasopharyngeal sample"[tiab:~2] OR "Nasopharyngeal samples"[tiab:~2] OR "Nasopharyngeal wash"[tiab:~2] OR "Nasopharyngeal washing"[tiab:~2] OR "Nasopharyngeal washes"[tiab:~2] OR "Naso pharyngeal swabbing"[tiab:~2] OR "Naso pharyngeal swabs"[tiab:~2] OR "Naso pharyngeal specimen"[tiab:~2] OR "Naso pharyngeal specimens"[tiab:~2] OR "Naso pharyngeal sample"[tiab:~2] OR "Naso pharyngeal samples"[tiab:~2] OR "Naso pharyngeal wash"[tiab:~2] OR "Naso pharyngeal washing"[tiab:~2] OR "Naso pharyngeal washes"[tiab:~2] OR "Respiratory swab"[tiab:~2] OR "Respiratory swabbing" [tiab:~2] OR "Respiratory swabs"[tiab:~2] OR "Respiratory specimen"[tiab:~2] OR "Respiratory specimens"[tiab:~2] OR "Respiratory sample"[tiab:~2] OR "Respiratory samples"[tiab:~2] OR "Nasopharyngeal aspirate"[tiab] OR "Naso pharyngeal aspirate"[tiab]

#7 “culture”[tiab] OR “cultivat*”[tiab] OR “isolat*”[tiab]

#8 "antigen detect*"[tiab] OR "antibody test*"[tiab] OR “immunoassay*”[tiab] OR "immuno assay*"[tiab] OR “immunofluorescen*”[tiab] OR "electron microscopy"[tiab] OR “ELISA”[tiab]

#9 #1 OR #2 OR #3 OR #4 OR #5 OR #6 OR #7 OR #8

#10 "chest infection"[tiab:~2] OR "chest infections"[tiab:~2] OR "chest infected"[tiab:~2] OR "respiratory infection"[tiab:~2] OR "respiratory infections"[tiab:~2] OR "respiratory infected"[tiab:~2] OR "lung infection"[tiab:~2] OR "lung infections"[tiab:~2] OR "lung infected"[tiab:~2] OR RTI[tiab] OR URTI[tiab] OR LRTI[tiab] OR "acute respiratory*"[tiab] OR "common cold*"[tiab]

#11 “viral*”[tiab] OR “virus*”[tiab] OR “virology”[tiab]

#12 “influenza*”[tiab] OR “flu”[tiab]

#13 “SARS-CoV-2”[tiab] OR “covid*”[tiab] OR “coronavirus*”[tiab] OR "severe acute respiratory syndrome*"[tiab] OR “SARS”[tiab] OR "Middle East respiratory syndrome*"[tiab] OR “MERS”[tiab]

#14 “picornavirus*”[tiab] OR “rhinovirus*” OR “parechovirus*”[tiab] OR “echovirus*”[tiab] OR “enterovirus*”[tiab]

#15 “Metapneumovirus*”[tiab] OR “HMPV”[tiab]

#16 “parainfluenza*”[tiab]

#17 “RSV”[tiab] OR "respiratory syncytial"[tiab]

#18 “Adenovirus*”[tiab] OR “Adenoviridae”[tiab]

#19 “herpes*”[tiab] OR "epstein-barr*"[tiab] OR “EBV”[tiab] OR “cytomegalovirus*”[tiab] OR “CMV”[tiab] OR "varicella"[tiab] OR “VZV”[tiab]

#20 "human bocavirus*"[tiab] or “HBoV”[tiab]

#21 #10 OR #11 OR #12 OR #13 OR #14 OR #15 OR #16 OR #17 OR #18 OR #19 OR #20

#22 “AMI”[tiab] OR “MI”[tiab] OR “STEMI”[tiab] OR "nSTEMI"[tiab]

#23 "coronary infarct"[tiab:~2] OR "coronary infarction"[tiab:~2] OR "coronary infarctions"[tiab:~2] OR "coronary ischaemia"[tiab:~2] OR "coronary ischemia"[tiab:~2] OR "coronary ischaemic"[tiab:~2] OR "coronary ischemic"[tiab:~2] OR "coronary attack"[tiab:~2] OR "coronary attacks"[tiab:~2] OR "cardiovascular infarct"[tiab:~2] OR "cardiovascular infarction"[tiab:~2] OR "cardiovascular infarctions"[tiab:~2] OR "cardiovascular ischaemia"[tiab:~2] OR "cardiovascular ischemia"[tiab:~2] OR "cardiovascular ischaemic"[tiab:~2] OR "cardiovascular ischemic"[tiab:~2] OR "cardiovascular attack"[tiab:~2] OR "cardiovascular attacks"[tiab:~2] OR "heart infarct"[tiab:~2] OR "heart infarction"[tiab:~2] OR "heart infarctions"[tiab:~2] OR "heart ischaemia"[tiab:~2] OR "heart ischemia"[tiab:~2] OR "heart ischaemic"[tiab:~2] OR "heart ischemic"[tiab:~2] OR "heart attack"[tiab:~2] OR "heart attacks"[tiab:~2] OR "myocardial infarct"[tiab:~2] OR "myocardial infarction"[tiab:~2] OR "myocardial infarctions"[tiab:~2] OR "myocardial ischaemia"[tiab:~2] OR "myocardial ischemia"[tiab:~2] OR "myocardial ischaemic"[tiab:~2] OR "myocardial ischemic"[tiab:~2] OR "myocardial attack"[tiab:~2] OR "myocardial attacks"[tiab:~2] OR "cardiac infarct"[tiab:~2] OR "cardiac infarction"[tiab:~2] OR "cardiac infarctions"[tiab:~2] OR "cardiac ischaemia"[tiab:~2] OR "cardiac ischemia"[tiab:~2] OR "cardiac ischaemic"[tiab:~2] OR "cardiac ischemic"[tiab:~2] OR "cardiac attack"[tiab:~2] OR "cardiac attacks"[tiab:~2]

#24 "ischaemic event"[tiab:~2] OR "ischaemic events"[tiab:~2] OR "ischemic event"[tiab:~2] OR "ischemic events"[tiab:~2] OR "ischaemia event"[tiab:~2] OR "ischaemia events"[tiab:~2] OR "ischemia events"[tiab:~2] OR "ischemia events"[tiab:~2] OR "thrombotic event"[tiab:~2] OR "thrombosis event"[tiab:~2] OR "thrombotic events"[tiab:~2] OR "thrombosis events"[tiab:~2]

#25 stroke*[tiab] OR "brain infarct"[tiab:~2] OR "brain infarction"[tiab:~2] OR "brain infarctions"[tiab:~2] OR "brain failure"[tiab:~2] OR "brain failures"[tiab:~2] OR "brain haemorrhage"[tiab:~2] OR "brain hemorrhage"[tiab:~2] OR "cerebral infarct"[tiab:~2] OR "cerebral infarction"[tiab:~2] OR "cerebral infarctions"[tiab:~2] OR "cerebral failure"[tiab:~2] OR "cerebral failures"[tiab:~2] OR "cerebral stroke"[tiab:~2] OR "cerebral strokes"[tiab:~2] OR "cerebrovascular infarct"[tiab:~2] OR "cerebrovascular infarction"[tiab:~2] OR "cerebrovascular infarctions"[tiab:~2] OR "cerebrovascular failure"[tiab:~2] OR "cerebrovascular failures"[tiab:~2] OR "cerebrovascular stroke"[tiab:~2] OR "cerebrovascular strokes"[tiab:~2] OR "ischaemic infarct"[tiab:~2] OR "ischaemic infarction"[tiab:~2] OR "ischaemic infarctions"[tiab:~2] OR "ischaemic failure"[tiab:~2] OR "ischaemic failures"[tiab:~2] OR "ischaemic stroke"[tiab:~2] OR "ischaemic strokes"[tiab:~2] OR "ischemic infarct"[tiab:~2] OR "ischemic infarction"[tiab:~2] OR "ischemic infarctions"[tiab:~2] OR "ischemic failure"[tiab:~2] OR "ischemic failures"[tiab:~2] OR "ischemic stroke"[tiab:~2] OR "ischemic strokes"[tiab:~2] OR "haemorrhagic infarct"[tiab:~2] OR "haemorrhagic infarction"[tiab:~2] OR "haemorrhagic infarctions"[tiab:~2] OR "haemorrhagic failure"[tiab:~2] OR "haemorrhagic failures"[tiab:~2] OR "haemorrhagic stroke"[tiab:~2] OR "haemorrhagic strokes"[tiab:~2] OR "hemorrhagic infarct"[tiab:~2] OR "hemorrhagic infarction"[tiab:~2] OR "hemorrhagic infarctions"[tiab:~2] OR "hemorrhagic failure"[tiab:~2] OR "hemorrhagic failures"[tiab:~2] OR "hemorrhagic stroke"[tiab:~2] OR "hemorrhagic strokes"[tiab:~2] OR "haemorrhage infarct"[tiab:~2] OR "haemorrhage infarction"[tiab:~2] OR "haemorrhage infarctions"[tiab:~2] OR "haemorrhage failure"[tiab:~2] OR "haemorrhage failures"[tiab:~2] OR "haemorrhage stroke"[tiab:~2] OR "haemorrhage strokes"[tiab:~2] OR "hemorrhage infarct"[tiab:~2] OR "hemorrhage infarction"[tiab:~2] OR "hemorrhage infarctions"[tiab:~2] OR "hemorrhage stroke"[tiab:~2] OR "hemorrhage strokes"[tiab:~2]

#26 "cardiovascular acute"[tiab:~1] OR "cardiovascular mortality"[tiab:~1] OR "cardiovascular mortalities"[tiab:~1] OR "cardiovascular death"[tiab:~1] OR "cardiovascular deaths"[tiab:~1] OR "cerebral acute"[tiab:~1] OR "cerebral mortality"[tiab:~1] OR "cerebral death"[tiab:~1] OR "cerebral deaths"[tiab:~1] OR "cerebrovascular acute"[tiab:~1] OR "cerebrovascular mortality"[tiab:~1] OR "cerebrovascular mortalities"[tiab:~1] OR "cerebrovascular death"[tiab:~1] OR "cerebrovascular deaths"[tiab:~1]

#27 #22 OR #23 OR #24 OR #25 OR #26

#28 ALL FIELDS

NOTNLM OR publisher[sb] OR inprocess[sb] OR pubmednotmedline[sb] OR indatareview[sb] OR pubstatusaheadofprint

#29 (((((randomized controlled trial[Publication Type]) OR (random*[Title/Abstract] OR placebo*[Title/Abstract] OR single blind*[Title/Abstract] OR double blind*[Title/Abstract] OR triple blind*[Title/Abstract]) OR (retraction of publication[Publication Type] OR retracted publication[Publication Type])) NOT (((comment[Publication Type] OR editorial[Publication Type] OR meta-analysis[Publication Type] OR practice-guideline[Publication Type] OR review[Publication Type] OR letter[Publication Type]) NOT randomized controlled trial[Publication Type]) OR ((random sampl*[Title/Abstract] OR random digit*[Title/Abstract] OR random effect*[Title/Abstract] OR random survey[Title/Abstract] OR random regression[Title/Abstract]) NOT randomized controlled trial[Publication Type]))) OR (cohort OR controlled-clinical-trial* OR epidemiolog*) OR (case* AND (control* OR series OR crossover OR cross-over))) NOT (("Animal" OR "animals" OR "rat" OR "rats" OR "mouse" OR "mice" OR "rodent*" OR "swine" OR "porcine" OR "murine" OR "sheep" OR "lamb" OR "lambs" OR "pig" OR "pigs" OR "piglet" OR "piglets" OR "rabbit" OR "rabbits" OR "cat" OR "cats" OR "dog" OR "dogs" OR "cattle" OR "bovine" OR "monkey" OR "monkeys" OR "trout" OR "marmoset" OR "marmosets") NOT ("human" OR "humans" OR "patient" OR "patients" OR "newborn*" OR "baby" OR "babies" OR "neonat*" OR "infan*" OR "toddler*" OR "pre-schooler*" OR "preschooler*" OR "kindergarten" OR "boy" OR "boys" OR "girl" OR "girls" OR "child" OR "children" OR "childhood" OR "adolescen*" OR "pediatric*" OR "paediatric*" OR "youth*" OR "teen" OR "teens" OR "teenage*" OR "school-aged*" OR "school-child*" OR "school-girl*" OR "school-boy*" OR "schoolgirl*" OR "schoolboy*" OR "man" OR "men" OR "woman" OR "women" OR "adult" OR "adults" OR "middle-age*" OR "elderly")))

#30 #9 AND #21 AND #27 AND #28 AND #29

**EMBASE (via Ovid) Search strategy**

1. (Lab* adj2 (test* or confirm* or result* or detect* or diagnos*)).tw,kf,dq.

2. exp Polymerase Chain Reaction/

3. (polymerase-chain-reaction* or PCR or multi-plex or multiplex or monoplex).tw,kf,dq.

4. ((Molecular or nucleic-acid or microbiologic*) adj1 (diagnos* or test* or assay*)).tw,kf,dq.

5. exp serology/ or exp microbiological examination/ or exp fluorescent antibody technique/

6. (serum* or serolog* or blood-sample or (sero* adj1 (positive* or negative* or epidemiolog*))).tw,kf,dq.

7. (respiratory-panel* or pathogen-panel* or RP-panel* or respiratory-pathogen*).tw,kf,dq.

8. (((throat* or nasal* or nasopharyngeal or naso-pharyngeal or respiratory) adj2 (swab* or specimen* or sample* or wash*)) or nasopharyngeal-aspirate or naso-pharyngeal-aspirate).tw,kf,dq.

9. (culture* or cultivat* or isolat*).tw,kf,dq.

10. (antigen-detect* or antibody-test* or immunoassay* or immuno-assay* or immunofluorescen* or immuno-fluorescen* or electron-microscopy or ELISA).tw,kf,dq.

11. or/1-10

12. *respiratory tract infection/ or exp *lower respiratory tract infection/ or exp *upper respiratory tract infection/ or exp *viral respiratory tract infection/

13. (((chest or respiratory or lung) adj2 infect*) or RTI or URTI or LRTI or acute-respiratory or common-cold*).tw,kf,dq.

14. exp *virus infection/ or exp *virus/

15. (viral* or virus* or virology).tw,kf,dq.

16. *influenza/ or exp *influenza a/ or *influenza b/ or *influenza pneumonia/ or exp *pandemic influenza/ or *seasonal influenza/

17. (influenza* or flu).tw,kf,dq.

18. *coronaviridae/ or exp *coronavirinae/ or exp *Coronavirus infection/

19. (SARS-CoV-2 or covid* or coronavirus* or severe-acute-respiratory-syndrome* or SARS or Middle-East-respiratory-syndrome* or MERS).tw,kf,dq.

20. exp *picornaviridae/ or exp *picornavirus infection/

21. (picornavirus* or rhinovirus* or parechovirus* or echovirus* or enterovirus*).tw,kf,dq.

22. exp *paramyxoviridae/ or exp *paramyxovirus infection/

23. exp *human metapneumovirus/ or exp *human metapneumovirus infection/

24. (Metapneumovirus* or HMPV).tw,kf,dq.

25. *respirovirus/ or *human parainfluenza virus 1/ or *human parainfluenza virus 3/ or exp *Parainfluenza virus infection/

26. parainfluenza*.tw,kf,dq.

27. exp *Human respiratory syncytial virus/ or exp *respiratory syncytial virus infection/

28. (RSV or respiratory-syncytial).tw,kf,dq.

29. exp *adenoviridae/ or exp *human adenovirus infection/

30. (Adenovirus* or adenoviridae).tw,kf,dq.

31. exp *herpesviridae/ or exp *herpes virus infection/

32. (herpes* or epstein-barr* or EBV or cytomegalovirus* or CMV or varicella or VZV).tw,kf,dq.

33. exp *bocaparvovirus/ or exp *Bocavirus infection/

34. (human-bocavirus* or HBoV).tw,kf,dq.

35. or/12-34

36. exp heart infarction/

37. (AMI or MI or STEMI or nSTEMI).tw,kf,dq.

38. ((coronary or cardiovascular or heart or myocardial or cardiac) adj2 (infarct* or isch?emi* or attack*)).tw,kf,dq.

39. ((isch?emi* or thrombo*) adj2 event*).tw,kf,dq.

40. exp cerebrovascular accident/ or exp brain hemorrhage/ or exp brain infarction/ or exp brain ischemia/

41. (stroke* or ((brain or cerebr* or isch?emi* or h?emorrhag*) adj2 (stroke* or infarct* or failure*))).tw,kf,dq.

42. ((cardiovascular or coronary or cerebrovascular) adj1 (mortalit* or death*)).tw,kf,dq.

43. or/36-42

44. (random$ or placebo$ or single blind$ or double blind$ or triple blind$).ti,ab.

45. RETRACTED ARTICLE/

46. or/44-45

47. (book or conference paper or editorial or letter or review).pt. not exp randomized controlled trial/

48. (random sampl$ or random digit$ or random effect$ or random survey or random regression).ti,ab. not exp randomized controlled trial/

49. 46 not (47 or 48)

50. exp cohort analysis/

51. exp longitudinal study/

52. exp prospective study/

53. exp follow up/

54. cohort$.tw.

55. exp case control study/

56. limit 54 to yr=1966-1989

57. exp case-control studies/

58. (case$ and control$).tw.

59. exp case study/

60. (case$ and (series or crossover or cross-over)).tw.

61. or/50-60

62. (rat or rats or mouse or mice or rodent* or swine or porcine or murine or sheep or lamb or lambs or pig or pigs or piglet or piglets or rabbit or rabbits or cat or cats or dog or dogs or cattle or bovine or monkey or monkeys or trout or marmoset or marmosets).ti. and animal experiment/

63. Animal experiment/ not (human experiment/ or human/)

64. or/62-63

65. 11 and 35 and 43 and (49 or 61)

66. 65 not 64

**Cochrane Library Central Register of Controlled Trials (CENTRAL)**

1 (Lab*:ti,ab,kw NEAR/2 (test*:ti,ab,kw OR confirm*:ti,ab,kw OR result*:ti,ab,kw OR detect*:ti,ab,kw OR diagnos*:ti,ab,kw))

2 [mh "Polymerase Chain Reaction"]

3 (polymerase-chain-reaction*:ti,ab,kw OR PCR:ti,ab,kw OR RT-PCR:ti,ab,kw OR multi-plex:ti,ab,kw OR multiplex:ti,ab,kw OR monoplex:ti,ab,kw)

4 ((Molecular:ti,ab,kw OR nucleic-acid:ti,ab,kw OR microbiologic*:ti,ab,kw) NEAR/1 (diagnos*:ti,ab,kw OR test*:ti,ab,kw OR assay*:ti,ab,kw))

5 [mh "Serologic Tests"] OR [mh "microbiological techniques"] OR [mh "fluorescent antibody technique"]

6 (serum*:ti,ab,kw OR serolog*:ti,ab,kw OR blood-sample:ti,ab,kw OR (sero*:ti,ab,kw NEAR/1 (positive*:ti,ab,kw OR negative*:ti,ab,kw OR epidemiolog*:ti,ab,kw)))

7 (respiratory-panel*:ti,ab,kw OR pathogen-panel*:ti,ab,kw OR RP-panel*:ti,ab,kw OR respiratory-pathogen:ti,ab,kw)

8 (((throat*:ti,ab,kw OR nasal*:ti,ab,kw OR nasopharyngeal:ti,ab,kw OR respiratory*:ti,ab,kw) NEAR/2 (swab*:ti,ab,kw OR specimen*:ti,ab,kw OR sample*:ti,ab,kw OR wash*:ti,ab,kw)) OR nasopharyngeal-aspirate:ti,ab,kw)

9 (culture*:ti,ab,kw OR cultivat*:ti,ab,kw OR isolat*:ti,ab,kw)

10 (antigen-detect*:ti,ab,kw OR antibody-test*:ti,ab,kw OR immunoassay*:ti,ab,kw OR immuno-assay*:ti,ab,kw OR immunofluorescen*:ti,ab,kw OR electron-microscopy:ti,ab,kw OR ELISA*:ti,ab,kw)

11 #1 OR #2 OR #3 OR #4 OR #5 OR #6 OR #7 OR #8 OR #9 OR #10

12 [mh "Respiratory Tract Infections"]

13 (((chest:ti,ab,kw OR respiratory:ti,ab,kw OR lung:ti,ab,kw) NEAR/2 infect*:ti,ab,kw) OR RTI:ti,ab,kw OR URTI:ti,ab,kw OR LRTI:ti,ab,kw OR acute-respiratory*:ti,ab,kw OR common-cold*:ti,ab,kw)

14 [mh "Virus Diseases"] OR [mh Viruses]

15 (viral*:ti,ab,kw OR virus*:ti,ab,kw OR virology:ti,ab,kw)

16 [mh "influenzavirus a"] OR [mh "influenzavirus b"] OR [mh ^"Influenza, Human"]

17 (influenza*:ti,ab,kw OR flu:ti,ab,kw)

18 [mh Coronaviridae] OR [mh "coronavirus infections"]

19 (SARS-CoV-2*:ti,ab,kw OR covid*:ti,ab,kw OR seasonal-coronavirus*:ti,ab,kw OR coronavirus*:ti,ab,kw OR severe-acute-respiratory-syndrome*:ti,ab,kw OR SARS:ti,ab,kw OR Middle-East-respiratory-syndrome*:ti,ab,kw OR MERS:ti,ab,kw)

20 [mh Picornaviridae] OR [mh "Picornaviridae Infections"]

21 (picornavirus*:ti,ab,kw OR (rhinovirus*or NEXT parechovirus*):ti,ab,kw OR echovirus*:ti,ab,kw OR enterovirus*:ti,ab,kw)

22 [mh Paramyxoviridae] OR [mh "Paramyxoviridae Infections"]

23 [mh ^Metapneumovirus] OR [mh "Pneumovirus Infections"]

24 (Metapneumovirus*:ti,ab,kw OR HMPV:ti,ab,kw)

25 [mh ^Parainfluenza] OR [mh "parainfluenza virus 1, human"] OR [mh "parainfluenza virus 3, human"]

26 parainfluenza*:ti,ab,kw

27 [mh ^"Respiratory Syncytial Virus, Human"] OR [mh ^"Respiratory Syncytial Virus Infections"]

28 (RSV:ti,ab,kw OR respiratory-syncytial*:ti,ab,kw)

29 [mh Adenoviridae] OR [mh "Adenovirus Infections, Human"]

30 Adenovirus*:ti,ab,kw

31 [mh Herpesviridae] OR [mh "Herpesviridae Infections"]

32 (herpes*:ti,ab,kw OR epstein-barr*:ti,ab,kw OR EBV:ti,ab,kw OR cytomegalovirus*:ti,ab,kw OR CMV:ti,ab,kw OR varicella-zoster:ti,ab,kw OR varicella-virus*:ti,ab,kw OR VZV:ti,ab,kw)

33 [mh ^"Human bocavirus"] OR [mh "Parvoviridae Infections"]

34 (human-bocavirus*:ti,ab,kw OR HBoV:ti,ab,kw)

35 #12 OR #13 OR #14 OR #15 OR #16 OR #17 OR #18 OR #19 OR #20 OR #21 OR #22 OR #23 OR #24 OR #25 OR #26 OR #27 OR #28 OR #29 OR #30 OR #31 OR #32 OR #33 OR #34

36 [mh "Myocardial Infarction"]

37 (AMI:ti,ab,kw OR MI:ti,ab,kw OR STEMI:ti,ab,kw OR non-STEMI:ti,ab,kw)

38 ((coronary:ti,ab,kw OR cardiovascular:ti,ab,kw OR heart:ti,ab,kw OR myocardial:ti,ab,kw OR cardiac:ti,ab,kw) NEAR/2 (infarct*:ti,ab,kw OR isch?emi*:ti,ab,kw OR attack*:ti,ab,kw))

39 ((isch?emi*:ti,ab,kw OR thrombo*:ti,ab,kw) NEAR/2 event*:ti,ab,kw)

40 [mh Stroke] OR [mh "Brain Infarction"] OR [mh ^"Cerebral Hemorrhage"] OR [mh "Subarachnoid Haemorrhage"]

41 (stroke*:ti,ab,kw OR ((brain:ti,ab,kw OR cerebr*:ti,ab,kw OR isch?emi*:ti,ab,kw OR h?emorrhag*:ti,ab,kw) NEAR/2 (stroke*:ti,ab,kw OR infarct*:ti,ab,kw OR failure*:ti,ab,kw)))

42 ((cardiovascular:ti,ab,kw OR cerebr*:ti,ab,kw) NEAR/1 (acute:ti,ab,kw OR mortality:ti,ab,kw OR death*:ti,ab,kw))

43 #36 OR #37 OR #38 OR #39 OR #40 OR #41 OR #42

44 #11 AND #35 AND #43 in Trials

**Web of Science Core Collection (Clarivate Analytics)**

#1 Lab* NEAR/2 (test* or confirm* or result* or detect* or diagnos*)

#2 polymerase-chain-reaction* or PCR or multi-plex or multiplex or monoplex

#3 (Molecular or nucleic-acid or microbiologic*) NEAR/1 (diagnos* or test* or assay*)

#4 serum* or serolog* or blood-sample or (sero* NEAR/1 (positive* or negative* or epidemiolog*))

#5 respiratory-panel* or pathogen-panel* or RP-panel* or respiratory-pathogen*

#6 (throat* or nasal* or nasopharyngeal or naso-pharyngeal or respiratory*) NEAR/2 (swab* or specimen* or sample* or wash*) or nasopharyngeal-aspirate or naso-pharyngeal-aspirate

#7 culture* or cultivat* or isolat*

#8. antigen-detect* or antibody-test* or immunoassay* or immuno-assay* or immunofluorescen* or immuno-fluorescen* or electron-microscopy or ELISA

#9 #1 OR #2 OR #3 OR #4 OR #5 OR #6 OR #7 OR #8

#10 ((chest or respiratory or lung) NEAR/2 infect*) or RTI or URTI or LRTI or acute-respiratory or common-cold*

#11 viral* or virus* or virology

#12 influenza* or flu

#13 SARS-CoV-2 or covid* or coronavirus* or severe-acute-respiratory-syndrome* or SARS or Middle-East-respiratory-syndrome* or MERS

#14 picornavirus* or rhinovirus*or parechovirus* or echovirus* or enterovirus*

#15 Metapneumovirus* or HMPV

#16 parainfluenza*

#17 RSV or respiratory-syncytial*

#18 Adenovirus* or adenoviridae

#19 herpes* or epstein-barr* or EBV or cytomegalovirus* or CMV or varicella or VZV

#20 #10 OR #11 OR #12 OR #13 OR #14 OR #15 OR #16 OR #17 OR #18 OR #19

#21 AMI or MI or STEMI or nSTEMI

#22 (coronary or cardiovascular or heart or myocardial or cardiac) NEAR/2 (infarct* or isch$emi* or attack*)

#23 stroke* or ((brain or cerebr* or isch$emi* or h$emorrhag*) NEAR/2 (stroke* or infarct* or failure*))

#24 (cardiovascular or cerebr*) NEAR/1 (acute or mortalit* or death*)

#25 #21 OR #22 OR #23 OR #24

#26 random* or placebo* or single blind* or double blind* or triple blind* or retraction or retracted or cohort* or controlled-clinical-trial* or epidemiologic or (case and (control* or series or crossover or cross-over))

#27 #9 AND #20 AND #25 AND #26
