# Supplementary material for: Laboratory-confirmed respiratory viral infection triggers for acute myocardial infarction and stroke: Systematic review protocol
Source: PLoS One. 2024 Jul 10;19(7):e0302748. doi: 10.1371/journal.pone.0302748 (PMC11236192; doi:10.1371/journal.pone.0302748)
Supplement: Attachment — Submitted filename: Response to Reviewers.docx [file pone.0302748.s003.docx]

**PLOS ONE**

**Manuscript ID: PONE-D-24-14603**

**Journal requirements**

| *1. Please ensure that your manuscript meets PLOS ONE's style requirements, including those for file naming.* | Manuscript formatting has been checked for compliance with PLOS ONE style requirements. |
| --- | --- |
| *2. We note that the grant information you provided in the ‘Funding Information’ and ‘Financial Disclosure’ sections do not match.*  *When you resubmit, please ensure that you provide the correct grant numbers for the awards you received for your study in the ‘Funding Information’ section.* | The funding sources added to ‘Funding Information’ have been corrected and now match the ‘Financial Disclosure’ section.  Award numbers are not applicable for these scholarships. |
| *3. Please review your reference list to ensure that it is complete and correct. If you have cited papers that have been retracted, please include the rationale for doing so in the manuscript text, or remove these references and replace them with relevant current references. Any changes to the reference list should be mentioned in the rebuttal letter that accompanies your revised manuscript. If you need to cite a retracted article, indicate the article’s retracted status in the References list and also include a citation and full reference for the retraction notice.* | References have been checked according to the NLM/ICMJE style. We have not made changes to the References list. |

**Response to Reviewers**

**Reviewer #1**

| *In line 107, authors must enter the protocol number registered with PROSPERO. In addition, they must correct repeated text: "for the effect of infection for the effect of infection", in line 155.* | Both these lines have been modified. |
| --- | --- |
| *Finally, before the conclusion, the authors must include a paragraph with potential limitations of the future systematic review, such as small number of studies, small samples in existing ones and expected biases in observational studies, explaining how they intend to deal with the limitations.* | Page 18-19, lines 347-357, limitations have been added prior to the conclusion paragraph. |

**Reviewer #2**

| *Abstract*  *Your abstract overall is very clear. Though you do describe a lot of publication-based eligibility criteria, the clinical criteria is kind of lacking (e.g. which infections, what tests, population groups, MI, stroke) which I think would bring added clarity.* | The abstract has been modified based on the reviewer’s suggestion to include clinical criteria (p. 3, lines 37-40). |
| --- | --- |
| *Methods*  *Love that you describe the changes from your registered protocol. I'm surprised though that these aren't addressed in this protocol rather than the published review. Unless I missed it? Or you mean *if* there are any amendments? Clarity here might be helpful.* | This sentence has been updated for clarity (p. 7, lines 109-111).  It now reads “If there are protocol amendments, important changes will be explained (including the date and reason for the amendment) in the completed published review, in addition to tracking in PROSPERO.” |
| *Your searches look excellent, thank you for providing all of them in full. I notice though that they aren't showing results line by line - this would be helpful to see as I wonder whether your study design filters are needed, since you're interested in many different study designs and adding these filters potentially adds bias to your search. If the difference in results is not great they may not be warranted.* | This review is ongoing. The study design filters used are justifiable given our preliminary results show they remove irrelevant citations allowing for better precision while remaining highly sensitive. As per PRISMA-P guidelines, the searches are included as part of the pre-determined search strategy. The final line by line search results will be provided with the completed published review.  This section in the text has been updated for brevity and clarity (p. 12, lines 216-218). |
| *You mention a librarian a few times in this protocol, and I see them acknowledged below. Did they write the search? If so, why are they not also an author on this project?* | While librarian has been invaluable and assisted in designing the search, however this would not constitute authorship as per ICMJE and as such we have included them in the acknowledgements. |
| *You could probably cut down the Search strategy section of the protocol, as it goes into an unnecessary level of detail.* | The search strategy section has been edited to remove redundant text (p. 12). |
| *Will the search for this review be peer-reviewed? Please describe.* | Our review does not include peer review of electronic search strategies. |
| *Data*  *Given PLoS' data policy, what are the team's plans for making data available such as full list of full-text exclusions, extraction data, and critical appraisal/quality checklists available?* | All relevant data from this study will be made available upon study completion as part of the manuscript or its supporting information, with the final published review. |
